# Manipulating the adhesion of electroless nickel-phosphorus film on silicon wafers by silane compound modification and rapid thermal annealing

**DOI:** 10.1038/s41598-017-08639-x

**Published:** 2017-08-29

**Authors:** Chin-Wei Hsu, Wei-Yen Wang, Kuan-Ting Wang, Hou-An Chen, Tzu-Chien Wei

**Affiliations:** Department of Chemical Engineering, National Tsing Hua University, Hsinchu, 30013 Taiwan

## Abstract

In this study, the effect of 3-2-(2-aminoethylamino) ethylamino propyl trimethoxysilane (ETAS) modification and post rapid thermal annealing (RTA) treatment on the adhesion of electroless plated nickel-phosphorus (ELP Ni-P) film on polyvinyl alcohol-capped palladium nanoclusters (PVA-Pd) catalyzed silicon wafers is systematically investigated. Characterized by pull-off adhesion, atomic force microscopy, X-ray spectroscopy and water contact angle, a time-dependent, three-staged ETAS grafting mechanism including islandish grafting, a self-assembly monolayer (SAM) and multi-layer grafting is proposed and this mechanism is well correlated to the pull-off adhesion of ELP Ni-P film. In the absence of RTA, the highest ELP Ni-P film adhesion occurs when ETAS modification approaches SAM, where insufficient or multi-layer ETAS grafting fails to provide satisfactory results. On the other hand, if RTA is applied, the best ELP Ni-P film adhesion happens when ETAS modification is islandish owing to the formation of nickel silicide, where SAM or multi-layer ETAS modification cannot provide satisfactory adhesion because the interaction between ETAS and PVA-Pd has been sabotaged during RTA. Evidenced by microstructural images, we also confirmed that ETAS can act as an efficient barrier layer for nickel diffusion to bulk silicon.

## Introduction

Electroless plating (ELP) of metal is a valuable technique for fabricating fine metal patterns on non-conducive substrates in microelectromechanical systems, circuit boards and the semiconductor industry^1–5^. In particular, the ELP of nickel-phosphorus (Ni-P) film has been a viable technique for preparing the barrier layer for copper diffusion in the IC industry. The principle of ELP involves a reduction of metal ions from the depositing bath to the substrate, which typically requires a catalyst such as palladium to lower the activation energy for metal nucleation. Because ELP occurs on the active sites of the catalysts, the adhesion of the deposited film, which is an important index for the reliability of the whole circuity, is highly related to the interaction between catalysts and the substrate^6–10^. In the past, adequate adhesion is obtained by either mechanically^11^ or chemically^11–13^ roughing the substrate before ELP so that the catalysts and the subsequent metal film can be firmly anchored. However, this strategy encounters big challenges nowadays because modern substrates are flat and are not allowed to roughen in above-mentioned manners in order to fulfill the requirement of ultra-fine linewidth and minimized signal loss.

To solve this urgent issue, one of the proposed methods is to modify the substrate surface with coupling agents. These coupling agents are usually equipped with a head group such as amine^14–16^, thiol^17–19^ or vinyl^20, 21^ and a tail group such as alkoxy (-OR)^22, 23^ or halogen (-Cl)^23^. Therefore, they can act bi-functionally: bonding the bottom substrate covalently through one group and interacting with the catalyst through another group, thus rendering noticeable improvement in the adhesion of the subsequent ELP film. Among the various coupling agents, amino-moiety containing silane-compounds such as 3-aminopropyltriethoxysilane(APTES),3-(2-Aminoethylamino)propyl]trimethoxysilane(EDAS) and 3- 2-(2-aminoethylamino) ethylamino propyl trimethoxysilane(ETAS) are widely studied owing to their ability to form a strong Si-O-Si covalent bond with any hydroxylated surface through their hydrolyzable alkoxy groups^24, 25^. In addition, their hydrophilic terminal groups are capable of interacting with noble metals owing to the excess of long pair electrons on the amino-moieties. In this way, the adhesion of ELP film can be strengthened.

We have previously synthesized polyvinyl alcohol-capped palladium nano-clusters (PVA-Pd) and applied them as the catalyst in the ELP Ni-P process on a 3- 2-(2-aminoethylamino) ethylamino propyl trimethoxysilane (ETAS)-modified Si surface. The above work proved that a strong donor-acceptor interaction exists between the Pd core of PVA-Pd and amino-moiety of ETAS. Lacking such strong interaction, commercial Sn/Pd catalysts fail to anchor on the ETAS-modified Si surface firmly and exhibit poor adhesion in subsequent ELP Ni-P film^10^. However, in our previous study, and according to our review of all relevant literature, the relation between ETAS modification and the adhesion of ELP film has never been explored. Most relevant studies have either reported the utilization of silane-compound for improving adhesion of deposited film^6–10^ or merely discussed how to control the morphology of the ETAS layer, without conducting ELP^15, 24, 25^. Very few reports have gone into details of how different levels of silane-compound modification influence the interfacial properties, and most importantly, how these different interfacial properties connect to the adhesion of the subsequent ELP layer. In this report, we aim to link molecular-scale ETAS modification to macroscopic properties like ELP film adhesion. In particular, several levels of ETAS modification on Si wafers were created by controlling the immersion time of ETAS stock solution; then these samples were carefully characterized by means of atomic force microscopy (AFM), water contact angle (WCA) and X-ray photoelectron spectroscopy (XPS) to understand the configurations of the ETAS layer. Finally, a scenario investigating ETAS modification on the Si wafer versus immersion time is proposed and the relation between ELP Ni-P film adhesion and ETAS configuration with or without post rapid thermal annealing (RTA) is discussed. To the best of our knowledge, this is the first report of this kind in the field of surface metallization, IC packing techniques and fine line circuits.

## Results and Discussion

### Pull-off Adhesion of ELP Ni-P Film

**Figure 1:**
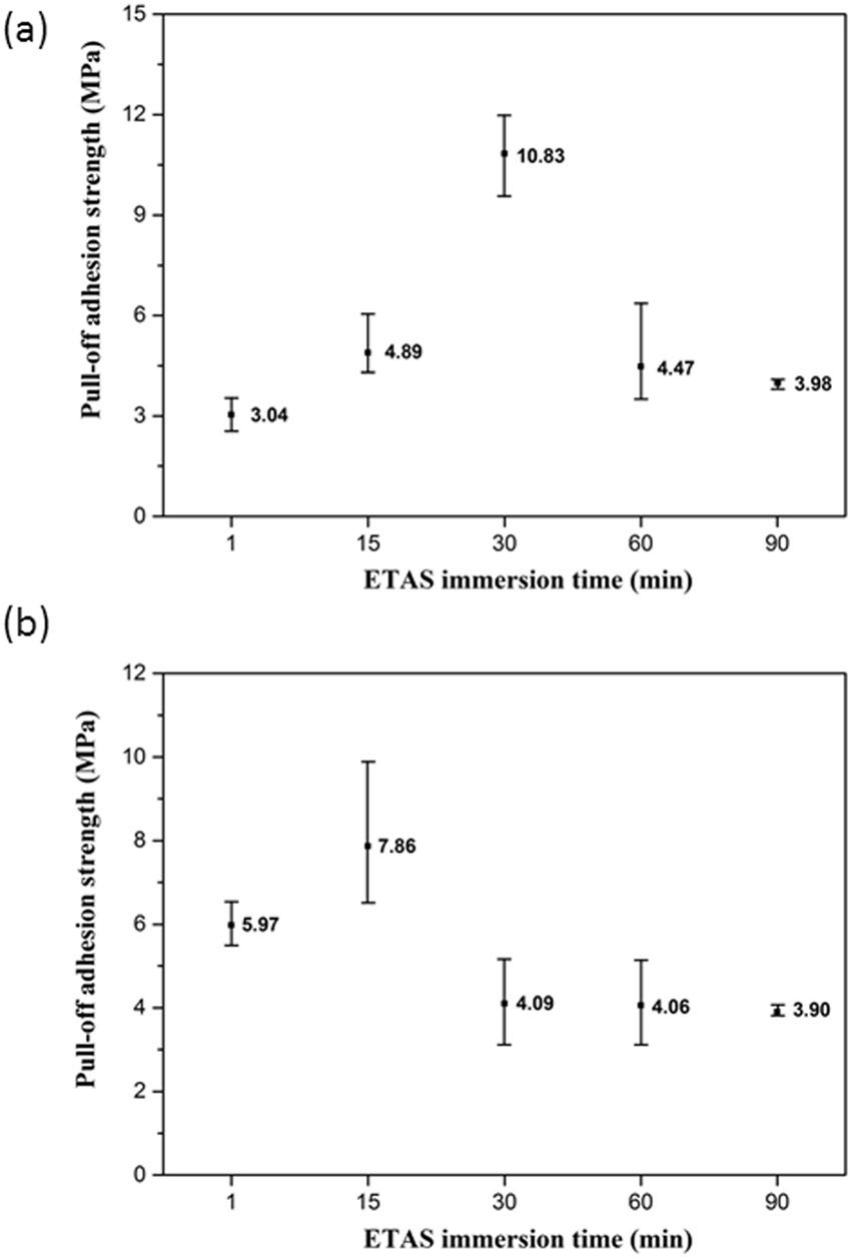
Pull-off adhesion test of ELP Ni-P film on Si wafer fabricated with different ETAS immersion from 1 to 90 min (**a**) before RTA, (**b**) after RTA. The value of each sample represents average pull-off adhesion strength of different ETAS immersion time from 1 min to 90 min.

Figure 1 depicts the measurement results of the adhesion of ELP Ni-P film on texturized Si wafers made with different ETAS immersion times. To obtain reliable accuracy, each condition contains 5 samples. From Fig. 1a, it can be seen that in the conditions without RTA, the average adhesion of ELP Ni-P film made with 1 minute of ETAS immersion (ETAS-1) is 3.04 MPa; the adhesion then increases to 4.89 MPa when ETAS immersion extends to 15 minutes (ETAS-15) and further increases to 10.83 MPa for 30 minutes of immersion time (ETAS-30). When ETAS immersion time lasts longer than 30 minutes, the adhesion decreases monotonically from 10.83 to 4.12 MPa for 60 minutes (ETAS-60) and to 3.57 MPa for 90 minutes (ETAS-90) of immersion. According to above result, it seems that the ETAS layer formed by 30 minutes of immersion is optimal in promoting the adhesion of ELP Ni-P film; this clue is very important in picturing the configuration of the ETAS layer in later discussion.

Interestingly, the average adhesion of ELP Ni-P films after RTA shows a different trend. As can be seen from Fig. 1b, the adhesion of ETAS-1 doubled from 3.04 to 5.97 MPa after RTA; this significant improvement is generally attributable to the formation of nickel-silicide between Ni-P and Si during RTA^26^, which is, in fact, one of the original purposes of RTA treatment. This intermetallic compound (IMC) joints the upper Ni-P film and bottom Si wafer and consequently reinforces the adhesion. For ETAS-15, the results verify that this effect is still valid with a profound enhancement of adhesion from 4.89 to 7.86 MPa. Surprisingly, for ETAS-30, the average adhesion dropped miserably from 10.83 to 4.09 MPa, representing a >50% decrease, contradicting the common sense of the purpose of RTA. Moreover, for ETAS-60 and ETAS-90, the adhesion stays at a low level of approximately 4 MPa after RTA, which is another uncommon result of RTA treatment.

### Morphological Characterization of an ETAS-modified Si Wafer

**Figure 2:**
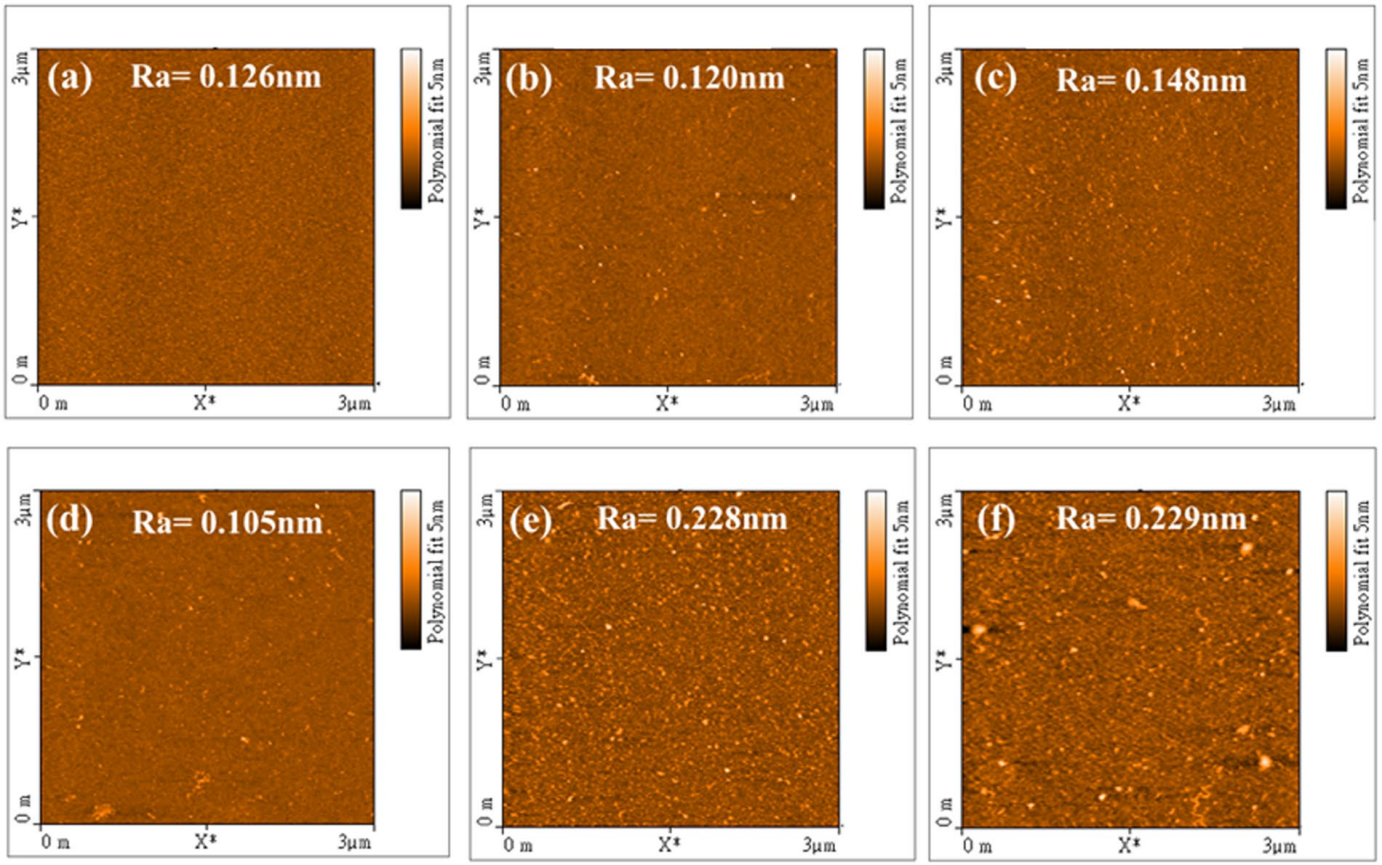
AFM images (3 × 3 μm^2^) of (**a**) bare polished Si wafer (without ETAS modification) and (**b**) ETAS-1, (**c**) ETAS-15, (**d**) ETAS-30, (**e**) ETAS-60 and (**f**) ETAS-90 modification.

To explain such dramatic change and more importantly, obtain insight into the role of the ETAS layer and the impact of RTA on the adhesion of ELP Ni-P film, several characterizations were conducted. AFM was firstly used to probe the morphological change on a polished Si wafer after ETAS modification. Figure 2 summarized the topographies of ETAS-modified Si wafers of different immersion times. Figure 2a is an image of a bare Si wafer. Because the bare wafer has been chemically polished before use, the surface is extremely smooth with an average roughness (Ra) of 0.126 nm. When ETAS was grafted on the wafer, small white spots were observed, as shown in Fig. 2b to f. Although these white spots are mostly like aggregated ETAS clusters rather than solo ETAS molecules because their visual sizes are much larger than an ETAS molecular in principal, these images still provide valuable information about the configuration of ETAS layer. This is because all AFM images revealed an identical roughness scale of 5 nm, indicating ETAS layers in all immersion times are comparably thin. As shown in Fig. 2b for ETAS-1, because the immersion time was so short, very few ETAS molecules were able to graft on the surface so that Ra kept almost unchanged. It should be emphasized that judging by the molecular length of an ideal ETAS, solely grafted ETAS cannot be resolved by AFM; the tiny white spots appearing in Fig. 2b should be aggregated ETAS clusters which were already formed in the IPA solution. In ETAS-15 (Fig. 2c), significant morphology change was observed as the number of white spots became much denser than they were in ETAS-1. These white spots are distributed quite uniformly, the Ra increased to 0.148 nm, meaning the Si surface became rougher after treatment with ETAS for 15 minutes; the result also implies that the configuration of ETAS-15 is islandish rather than a self-assembly monolayer (SAM) because of the increased Ra. When ETAS immersion time increased to 30 minutes (Fig. 2d), the topography changed again significantly because a considerable amount of white spots disappeared, accompanied by a very small R_a_ of 0.105 nm, which is even flatter than that of an untreated bare wafer. We attribute this change to the formation of SAM. Since AFM probes a sample’s morphology by tapping its surface, the fact of decreased Ra from Fig. 2c to 2d suggests that a continuous layer composed of identical soft substance (ETAS) was grafted on the wafer, resulting in a decreased Ra. In ETAS-60 and ETAS-90 shown in Fig. 2e and 2f, respectively, it can be seen that a lot of white spots re-appeared from the status of ETAS-30, suggesting that the Si surface was covered by many aggregated ETAS clusters. Closely comparing the sizes of white spots in Fig. 2c and 2e reveals that the sizes of white spots in Fig. 2e are more uniform but larger than those in Fig. 2c, hinting that the origin of the white spots in these two images is different. As mentioned above, white spots in Fig. 2c are attributed to aggregated ETAS clusters which already formed in the IPA solution. Therefore, their sizes are randomly determined. On the other hand, white spots in Fig. 2e are associated with ETAS grafting on the ETAS SAM, which formed in the ETAS-30 stage, rendering ETAS clusters with more uniform size. This effect became more obvious upon prolonged ETAS immersion in ETAS-90 as several large ETAS clusters up to 200 to 300 nm can be identified in Fig. 2f, showing that the aggregation of ETAS on the Si surface is time dependent and hence control of the ETAS immersion time is very important in determining the configuration of the ETAS layer. Owing to the formation of ETAS multi-layers in ETAS-60 and ETAS-90, the Si surface was roughened and Ra increased to 0.228 and 0.229 nm, respectively.

**Figure 3:**
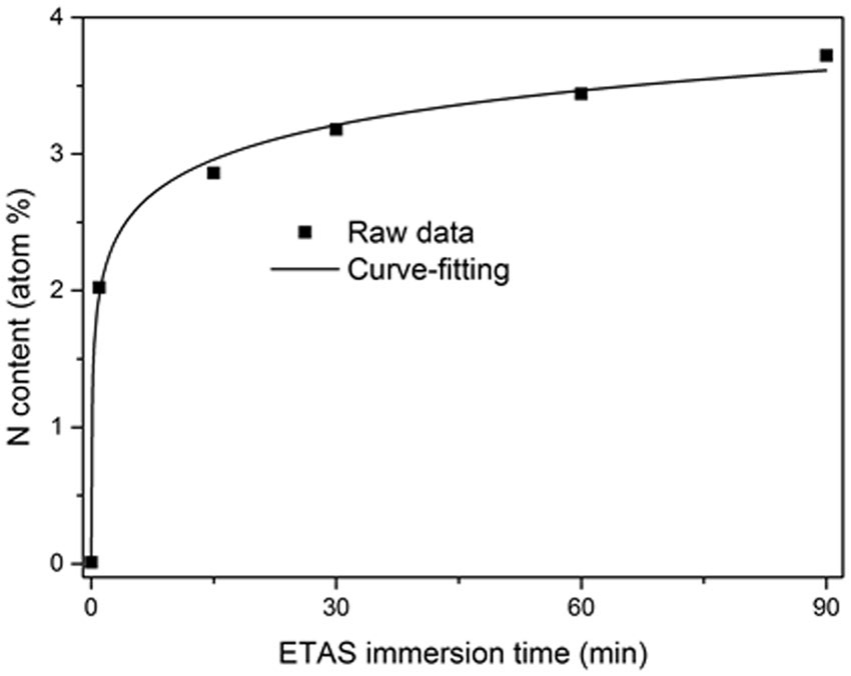
Nitrogen content of ETAS-modified Si surface determined by XPS versus various ETAS immersion time from 1 min to 90 min (1 min, 15 min, 30 min, 60 min and 90 min).

XPS was utilized to determine the amount of grafted ETAS molecules on the Si surface. Because ETAS is the only source of nitrogen atoms in the ETAS-modified Si wafer, we consequently report the nitrogen content by XPS analysis. This allows us to link to the amount of ETAS on the Si surface. Figure S1 shows XPS wide scans of ETAS-modified Si wafers. In Figure S1, it can be found the most significant change after ETAS modification is the appearance of N1s signal with a binding energy of approximately 400.7 eV, directly evidencing successful grafting of ETAS on the Si wafer in our approach. The element contents of oxygen, carbon, silicon and nitrogen from these spectrums were integrated and summarized in Table 1. The table shows that the nitrogen content increased rapidly from zero to 2.02%, 2.86% and 3.18% for ETAS-1, ETAS-15. ETAS-30, respectively, followed by a mild increase to 3.44% and 3.72% for ETAS-60 and ETAS-90, respectively. For a better presentation, the relation between nitrogen content and ETAS immersion time was plotted in Fig. 3. Interestingly, a turning point in the rate of nitrogen content increase was found at approximately 30 minutes of ETAS immersion time, implying the grafting of ETAS within 90 minutes involves two mechanisms and these two mechanism switched at the point of 30 minutes. At the initial stage of ETAS immersion, the nitrogen content increased sharply because ETAS molecules are grafted directly on the Si wafer; as immersion progresses, more and more of the Si surface is occupied by grafted-ETAS and this situation hinders subsequent grafting of ETAS molecules. The moment after SAM forms, new ETAS grafting becomes sluggish because tilting of ETAS molecules in the solution is needed to fit the favorable alignment with ETAS SAM, rendering slow growth in ETAS loading. From the result presented in Fig. 3, a turning point of nitrogen content at approximately 30 minutes of ETAS immersion time is clearly observed, meaning ETAS-30 mostly approaches the status of SAM formation.

**Table 1:** Atomic percent value of silicon surface obtained in XPS with different ETAS immersion time from 1 min to 90 min.

Element	1 min	15 min	30 min	60 min	90 min
O	33.37%	32.81%	32.08%	33.75%	33.29%
C	16.50%	18.11%	19.81%	20.2%	17.07%
Si	48.11%	46.21%	44.92%	42.61%	45.92%
N	2.02%	2.86%	3.18%	3.44%	3.72%

**Figure 4:**
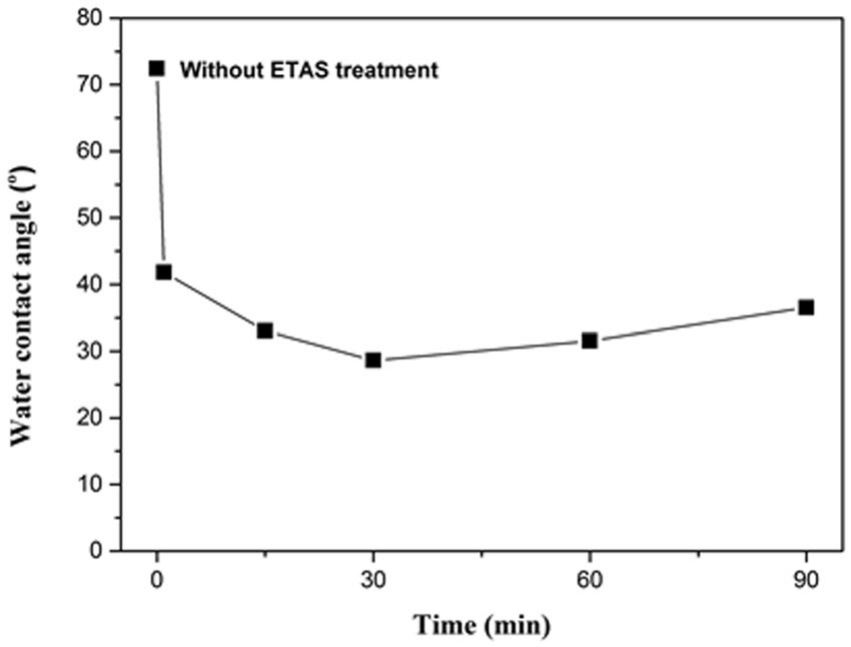
WCA on ETAS-modified Si surface versus different ETAS immersion time from 1 min to 90 min (1 min, 15 min, 30 min, 60 min and 90 min).

To verify this speculation, WCA was applied to examine the hydrophilicity of the ETAS-modified surface. Because amino-moiety is more hydrophilic than bare Si, the decrease of WCA after ETAS immersion is therefore evidence of ETAS grafting. The representative images of the WCA test are detailed in Figure S2 and the average WCA of 5 samples are plotted in Fig. 4. From Fig. 4, it can be seen the WCA of the bare wafer was 72.4°, which is typical for a polished Si wafer; for ETAS-1, WCA dropped significantly to 37.3°, suggesting ETAS molecules have grafted on Si surface and their hydrophilic amino-moieties are positioned upward. The surface becomes more and more hydrophilic under prolonged ETAS immersion. The lowest WCA of 28.6° was detected for ETAS-30, suggesting surface loading of upright-aligned amino-moiety was saturated and the configuration of the ETAS layer approached SAM. For ETAS-60 and ETAS-90, the WCA became 31.0° and 36.5°, respectively, meaning the surface became less hydrophilic than under the condition of ETAS-30. According to the XPS analysis discussed above, although the grafting of ETAS on the Si surface may involve a two-stage mechanism, the total ETAS amount is still positively proportional to immersion time; in other words, longer immersion time results in more ETAS grafting. Hence, the only possible cause of increased WCA when ETAS loading increases is that some ETAS molecules are not positioned as expected. From AFM observation and XPS analysis, ETAS-30 could approach SAM. For ETAS immersion over 30 minutes, ETAS molecules were grafted on the SAM of ETAS rather than on the Si surface. Amino moiety-containing silane compounds are known to undergo intra-molecule or inter-molecule aggregation because of their excess of long pair electrons on nitrogen atoms^25^, which leads to chaotic configuration of the capping ETAS layer over the bottom SAM. The chaos may include upside down, horizontally laid or even self-curled ETAS molecules, possibly exposing a few methoxy tails. These methoxy groups are less hydrophilic than amino moieties; consequently, WCA increased. Our data reveals change in WCA when above-described situation occurs. However, the change is not obvious because it fell within the range from 3° to 4°, suggesting the surface of the overall ETAS structure is still amino moiety-rich. Thus, the interactions between amino moieties and PVA-Pd and subsequent ELP are still valid.

### Time-dependent Grafting Mechanism of ETAS on Si Wafers

**Figure 5:**
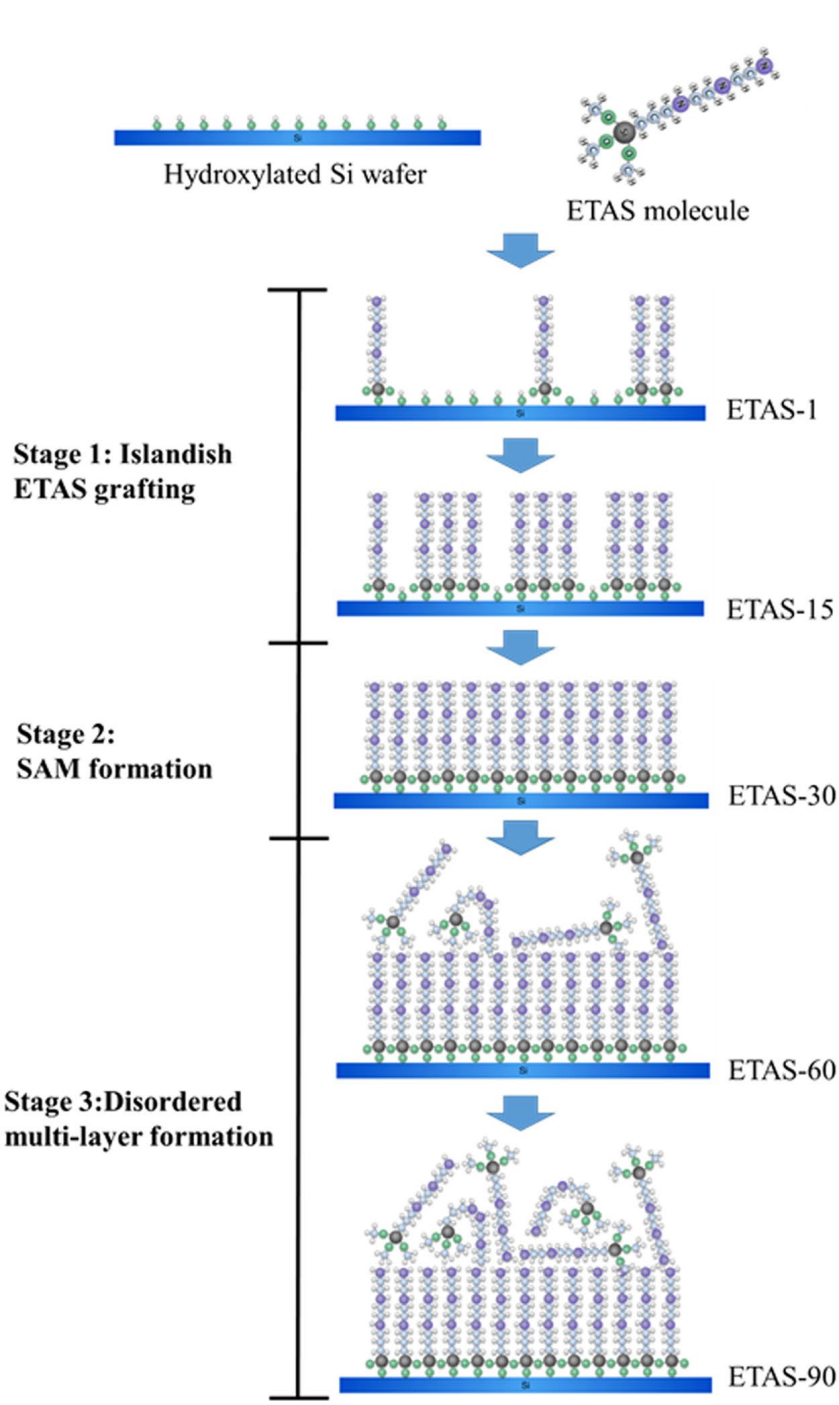
Imaginary illustration of time-dependent ETAS grafting mechanism on Si Wafer.

Combining the information obtained from AFM, XPS and WCA, a mechanism of ETAS grafting onto a Si wafer is proposed below and cartooned in Fig. 5:

**Stage 1, Islandish ETAS grafting:** When the ETAS immersion time is short, owing to insufficient time allowed for ETAS grafting, only a small portion of the Si surface is grafted by ETAS. These early-grafted ETAS molecules are separated from each other to maintain thermodynamic stability, rendering many isolated ETAS islands. The validation of this stage can be verified by microscopic morphology observation, such as AFM, or examination of macroscopic hydrophilicity change, such as WCA measurement.

**Stage 2, SAM formation:** When ETAS immersion time is long enough, ETAS is capable of grafting on the gap between early-grafted ETAS islands, gradually forming a SAM. The approaching of SAM formation can be determined when Ra becomes lower than that in stage 1 or even that of a raw substrate. In addition, in this stage the surface shows the lowest WCA due to the presence of the densest upright-aligned amino-moieties.

**Stage 3, Disordered multi-layer formation:** For prolonged immersion time after SAM formation, excess ETAS molecules are grafted onto the SAM surface in many orientations, forming a multi-layer ETAS structure with an ordered SAM in the bottom layer and a disordered surface layer. The junction of SAM with the disordered surface structure is weakly connected when compared with the covalently bonded junction between SAM and Si wafer. To verify the transition from Stage 2 to Stage 3, probing the slope of the increase of surface ETAS content against immersion time is an efficient method. Alternatively, monitoring the slight increase in WCA from Stage 2 also works.

### Correlation between ELP Ni-P Film Adhesion and ETAS Modification

The above-mentioned three-stage scenario is now applied to explain the dramatic change in the adhesion of ELP Ni-P after RTA. Without RTA, the strength of Ni-P film adhesion relies on the interaction between the amino-moieties of ETAS and the PVA-Pd catalyst^10^. This explains why ELP Ni-P film made under the ETAS-30 condition without RTA exhibits the best adhesion because the Si surface is covered by an ETAS SAM of ETAS. Less than 30 minutes of immersion (ETAS-1 and ETAS-15) results in weaker ELP Ni-P adhesion than ETAS-30 because there are less amino-moieties and consequently less capability to interact with PVA-Pd. As a result, the adhesion of subsequent ELP Ni-P film is poor. For ETAS-60 and ETAS-90, the surface is grafted by many ETAS molecules and therefore the number of adsorbed PVA-Pd should be abundant. Unfortunately, though, these upper ETAS molecules are grafted on ETAS SAM by weak forces such as a hydrogen bond or Van der Waals attraction rather than covalently bonded with Si as they are designed to be. Consequently, during the pull-off test, the weakest interface of the whole Si/ETAS SAM/disordered ETAS layer/PVA-Pd/ELP Ni-P structure, which is believed to be somewhere between ETAS SAM and the disordered ETAS layer, is torn. This mechanism also explains why ETAS-60 and ETAS-90 render similarly poor adhesion of ELP Ni-P film because once disordered ETAS layer is formed, somewhere in the disordered ETAS layer determines the adhesion, no matter how abundant or how thick the disordered ETAS layer is.

As mentioned above, the result of the pull-off adhesion test after RTA differs from the one without RTA significantly. After RTA, the adhesion of ETAS-1 and ETAS-15 increased by almost 2 fold; for ETAS-30, the adhesion dropped by 2.5 fold and for ETAS-60 and ETAS-90, the adhesion of ELP Ni-P film stayed at an unsatisfactorily low level of approximately 4 MPa. Coincidentally, above result has three categories, implying it is closely related to the three-stage ETAS grafting scenario.

**Figure 6:**
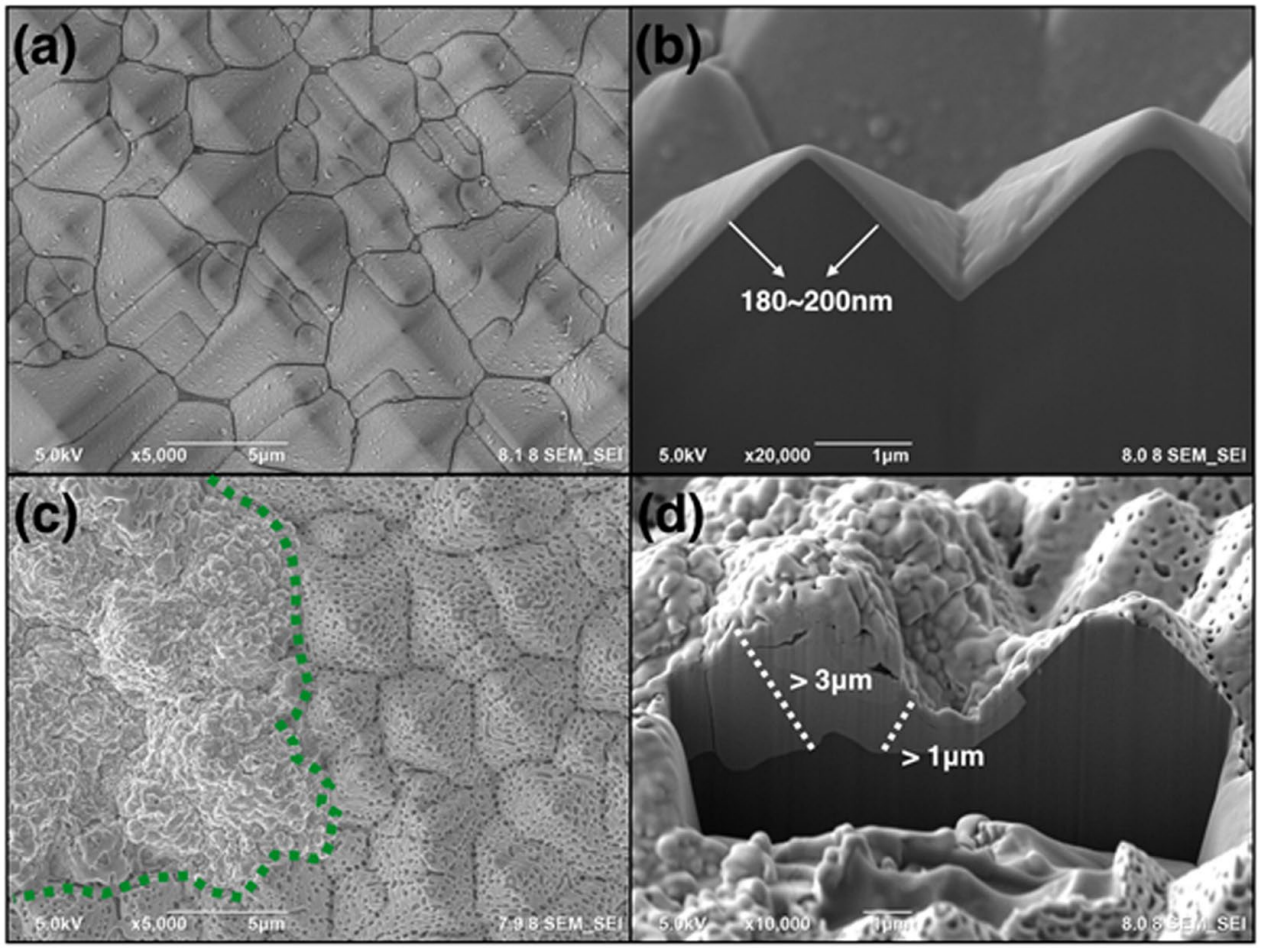
Topographical and cross-sectional SEM images of ELP Ni-P film on texturized Si wafer made by ETAS-15 modification before (**a**,**b**) and after (**c**,**d**) RTA. The dashes greed line indicates two different morphologies of ELP Ni-P film caused by RTA process.

**Figure 7:**
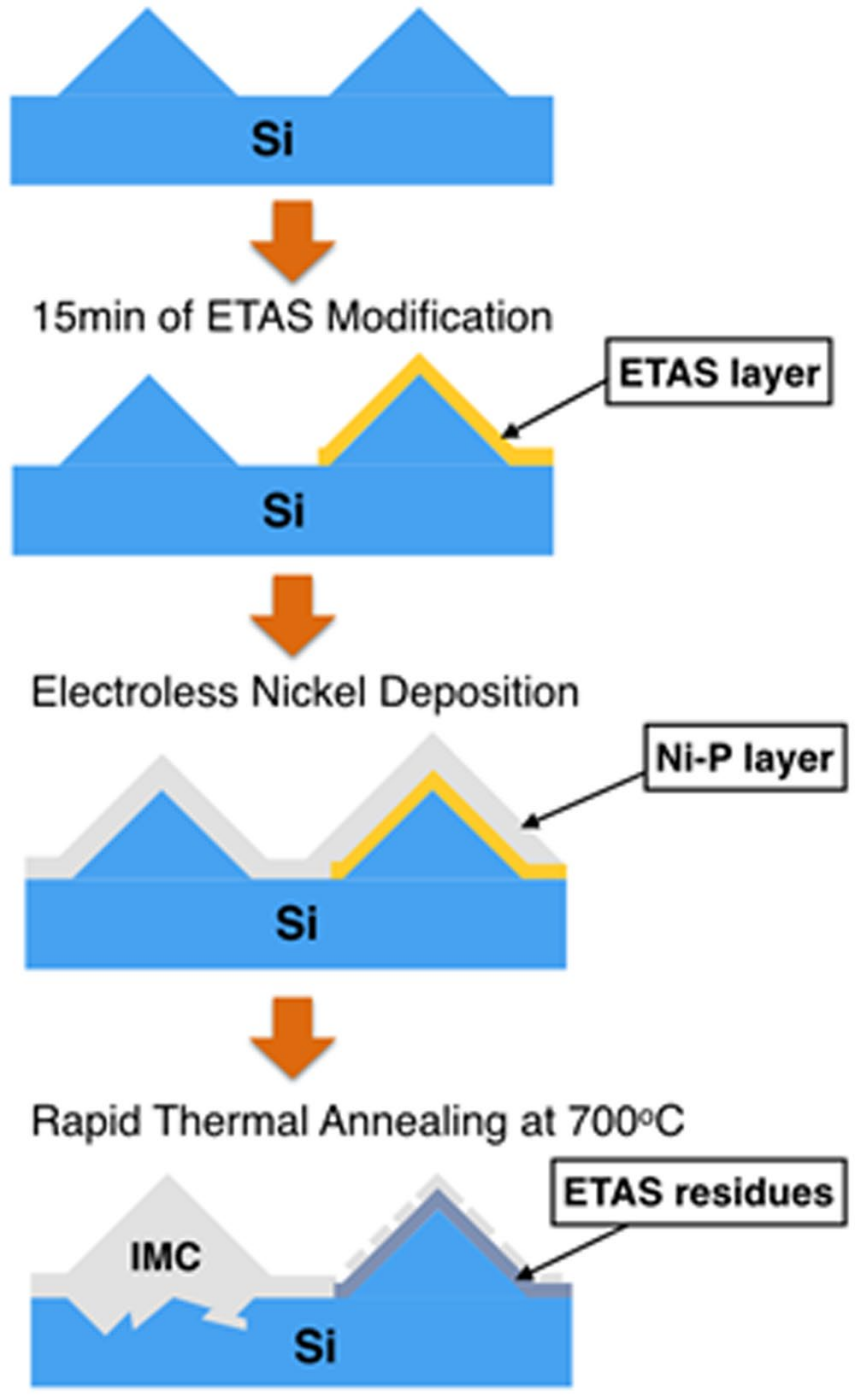
Formation mechanism of thick IMC from thin ELP Ni-P film in the case of islandish ETAS-grafting.

**Figure 8:**
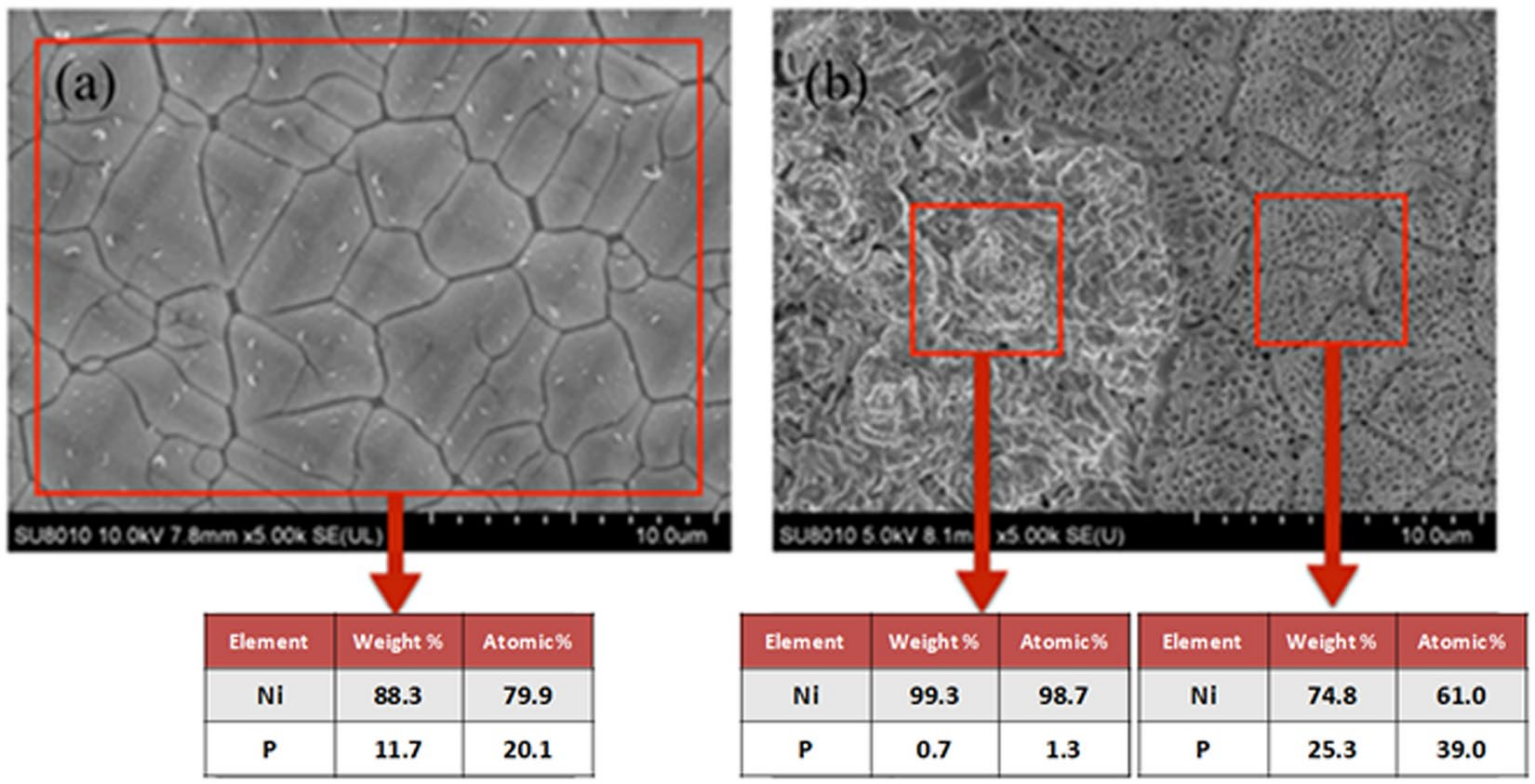
EDX analysis of ELP Ni-P film (**a**) before RTA and (**b**) after RTA on ETAS-15 modified, PVA-Pd activated texturized Si wafer.

During stage 1 of ETAS grafting, the ETAS layer is islandish. Therefore, part of the Si surface contacts ELP Ni-P film directly without being hindered by ETAS. The contact zone forms IMC during RTA and hence contributes considerable adhesion. Figure 6 is a topographical and cross-sectional view of ELP Ni-P on a texturized Si wafer made by ETAS-15 before (Fig. 6a and b) and after (Fig. 6c and d) RTA. As can be compared easily, the morphology of ELP Ni-P film changed enormously from smooth to uneven after RTA. The uneven surface in Fig. 6c can be further identified as a rugged section on the left side and a porous section on the right side. A dashed green line indicates the boundary between these two zones. Tilting Fig. 6a and c by 52 degrees, the cross-sectional view provides more evidences of our hypothesis. As can be seen clearly in Fig. 6b, ELP Ni-P film is deposited along the pyramidal surface perfectly. This is beneficial due to the strong donor-accepter interaction between the PVA-Pd catalyst and ETAS. The thickness of the ELP Ni-P layer is visually evaluated as approximately 200 nm, matching the value suggested by the vendors. After RTA, Fig. 6d shows that IMC formed selectively in the rugged zone, not in the porous zone. Surprisingly, the thickness of IMC exceeds 2μm, which is 10 times thicker than as-deposited ELP Ni-P film. On the other hand, the thickness of the porous zone stays unchanged at approximately 200 nm. To explain this bizarre thickness of IMC growth, we speculate that the configuration of the ETAS layer between the ELP Ni-P film and Si wafer plays an important role. Figure 7 cartoons the formation mechanism of 2μm-thick IMC from 200nm-thick ELP Ni-P: After ETAS-15 treatment, the surface of the Si wafer was partially grafted with ETAS clusters. After PVA-Pd catalyzing and the ELP process, the whole surface was deposited by a 200nm-thick Ni-P layer and since the ETAS layer was islandish, part of the ELP Ni-P film contacted the Si surface directly without ETAS lying in between. During RTA, the nickel of the Ni-P film diffused vertically into Si and formed nickel silicide from the direct-contact zone. The nickel above the ETAS-grafted Si first had to diffuse horizontally to the territory of the direct-contact zone and then formed IMC accordingly because of ETAS hindrance. In the direct-contact zone, the IMC layer grew unexpectedly thick because of this extra nickel supply; in the ETAS-grafted zone, the Ni-P film became hollow because of this diffusional loss of nickel. EDX elemental analysis echoes this speculation. Shown in Fig. 8, the atomic ratio of Ni:P in Ni-P film before RTA (Fig. 8a) is 79.9:20.1, perfectly matching the film composition suggested by the vendors. After RTA (Fig. 8b), the atomic ratio of Ni:P became 98.7:1.3 and 61.0:39.0 in the rugged and porous zones, respectively. Since phosphorus is stagnant during the formation of IMC, the increased phosphorus portion in the porous zone solidly indicates nickel migrated out of this zone. On the other hand, in the rugged zone, the highly pure nickel content suggests a significant amount of foreign nickel immigrated into this zone. Conclusively, without RTA, the adhesion of ELP Ni-P film relies on the interaction between ETAS and the PVA-Pd catalyst; after RTA, the adhesion of ELP Ni-P film relies on IMC. Apparently, the strength of IMC is larger than the interaction between ETAS and PVA-Pd and consequently the adhesion increased from 4.89 to 7.86 MPa.

**Figure 9:**
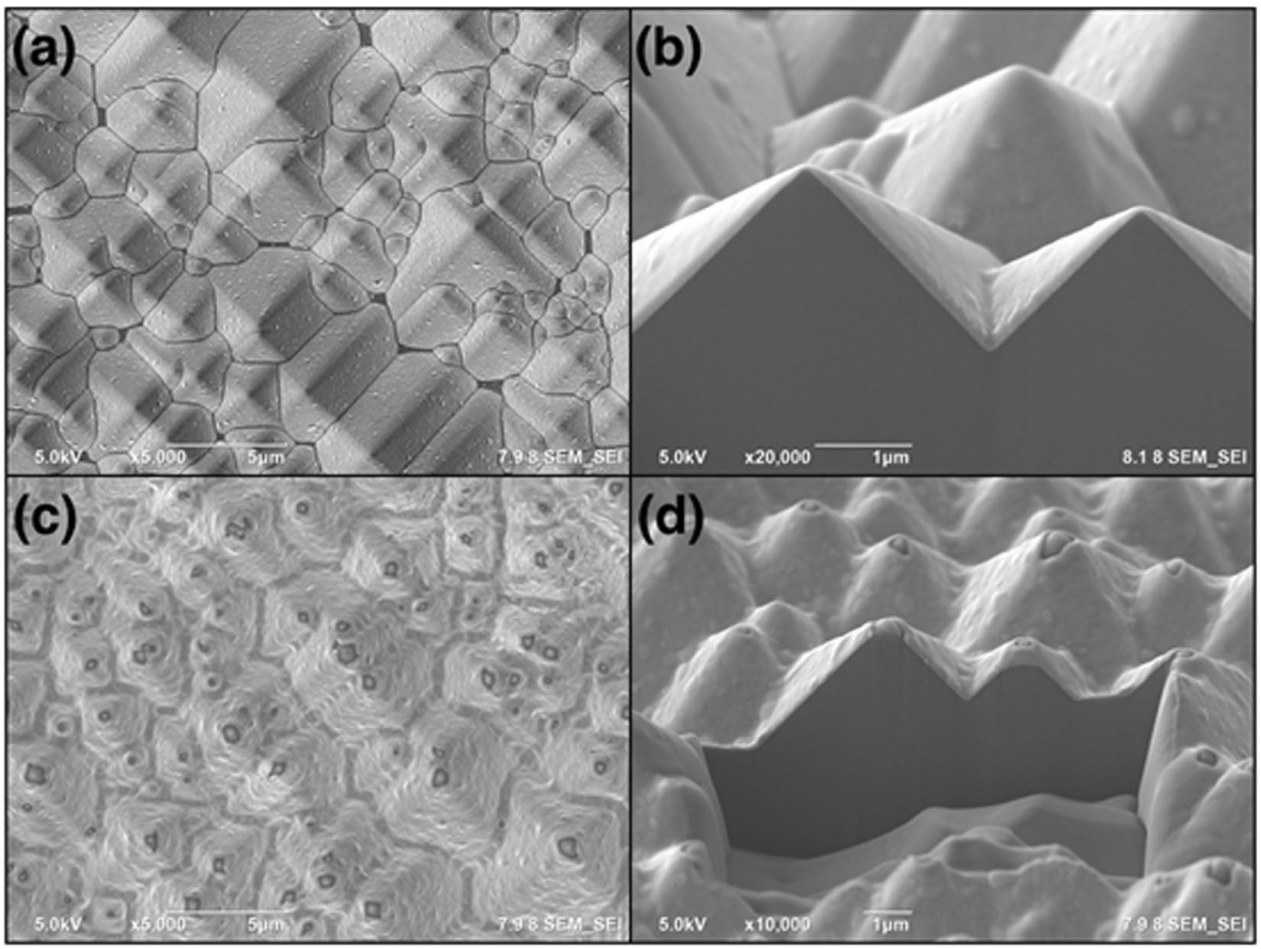
Topographical and cross-sectional SEM images of ELP Ni-P film on texturized Si wafer made by ETAS-30 modification before (**a**,**b**) and after (**c**,**d**) RTA.

Similar morphological analysis of ETAS-30 is shown in Fig. 9, in which Fig. 9a and b are topographical and cross-sectional SEM images of ELP Ni-P film made with ETAS-30 and without RTA, respectively. Both images resemble Fig. 6a and Fig. 6b because the configuration of ETAS modification is irresolvable by SEM. Fortunately, after RTA, Fig. 9c and d appear to be much different than their counterparts, which are Fig. 6c and d. The topography (Fig. 9c) of ELP Ni-P film made by ETAS-30 after RTA was slightly wrinkled but still homogeneous. A cross-sectional view (Fig. 9d) revealed that the boundary of the ELP Ni-P layer and Si remains distinct compared with Fig. 6d, indicating no IMC formation occurred in this sample. Krishnamoorthy *et al*.^27^ found silane-compound modification functions as a diffusion barrier for copper and silicon. As proved above, ETAS-30 formed SAM, and hence, the above-mentioned nickel diffusion into silicon, either vertically or horizontally, is prohibited by this ETAS SAM. Surprisingly, though the decomposing temperature of ETAS is 225 °C (Figure S3), it still provides incredible and nearly perfect isolation of Ni-P and Si at the 700 °C annealing condition. Unfortunately, although SAM ETAS served as an “invisible” diffusion barrier layer, it somehow lost its original purpose, which is bridging amino-moieties and Pd PVA-Pd because ETAS was definitely decomposed during RTA and only carbon-based residues were left in the interface, resulting in a dramatic decrease of adhesion. Similar results were also found in the samples of ETAS-60 and ETAS-90. Because ETAS molecules lost their bridging capability during RTA, the Ni-P adhesion of ETAS-30, ETAS-60 and ETAS-90 converged to the same level of approximately 4 MPa, whether they were SAM or multi-layer prior RTA treatment.

## Conclusions

In conclusion, we systematically investigated the effect of ETAS immersion time and RTA treatment on the adhesion of ELP Ni-P film on texturized Si wafers. It is concluded that if RTA is unnecessary or not allowed in the entire metallization process, forming a SAM during silane-compound modification and then catalyzing by PVA-Pd are keys to develop a highly adhesive ELP Ni-P layer. On the other hand, if RTA is necessary or inevitable throughout the entire metallization process, forming an islandish silane-compound coating is favorable in promoting the adhesion of ELP Ni-P film because an islandish silane-compound layer allows the formation of IMC during RTA. Moreover, in this study, we disclose various techniques such as AFM, WCA and the pull-off adhesion test to determine the silane compound configuration, which is usually in the scale of a few nanometers. Future applications can develop by utilizing the three-stage grafting scenario proposed in this study on various substrates such as glass, ceramics and so on, aiming to manipulate interfacial properties to meet modern metallization requirements.

## Methods

A KOH texturized n-type Si wafer (62.4 Ω/square, Gintech Corporation, Taiwan) was used as the substrate in this study. The Si wafer was cleaned by the RCA (Radio Corporation of America) process, followed by dipping in an aqueous solution containing 2% HF sequentially to remove organic species and native oxide on the wafer, respectively. When HF treatment is completed, the Si wafer is subject to rinse by ultrapure water to remove possible HF residues and create surface oxide layer. After water rinsing, the Si wafer was immersed in pure isopropanol (IPA, 99.5%, Macron, USA) first to remove possible water residue and then immersed in an isopropanol (IPA, 99.5%, Macron, USA) solution containing 1 vol% ETAS ( >95%, Acros, USA). The immersion time varied from 1 to 90 minutes in order to control the level of silanization. It should be noted that after ETAS immersion, the ETAS-attached Si wafer was sonicated in pure IPA for 5 minutes. The purpose of this treatment was to remove weakly-attached ETAS. Finally, the sample was baked in an oven at 120 °C for 30 minutes to form the Si-O-Si covalent bond and remove IPA traces simultaneously. ETAS-modified Si wafers were characterized by AFM (Nanosurf, C3000, Switzerland) to examine their topographical images, WCA (SEO, Phoenix-I, Korea) to observe their surface hydrophilicity and XPS (VGS, Thermo K-Alpha, USA) to determine the ETAS loading on the Si wafer. Aqueous polyvinyl alcohol-capped Pd nanoclusters (PVA-Pd) colloids were synthesized according to a procedure published elsewhere^28^ and diluted to 50 ppm with deionized water. To adsorb catalysts, the above-prepared silanized Si wafer was immersed in PVA-Pd solution for 5 minutes at 40 °C, followed by rinsing with deionized water and drying at ambient temperature.

Ni-P film was then ELP on the PVA-Pd attached, ETAS-modified Si wafer in a commercial bath (9026 M, OMG, USA) at 80 °C for 1 minute. The thickness of as-deposited Ni-P film was measured to be 200 nm by cross-sectional scanning electron microscopy (SEM, JEOL, JSM-5600) image (shown in Figure S4); this finding is consistent with the thickness of Ni-P ELP film made by commercial Sn/Pd catalyst, indicating the ELP rate stays unchanged despite the use of different catalysts. The as-deposited samples may be subjected to rapid thermal annealing (RTA, OLYMPUS, e1200-RTP) in N_2_ atmosphere at 700 °C for 2 minutes. The mechanical adhesion of ELP Ni-P film was quantitatively examined by a pull-off adhesion tester (PosiTest AT-M, DeFelsko, USA), which complies with ASTM D4541/D7234. The testing procedure involves sticking an aluminum dolly onto as-deposited ELP Ni-P film with a thermally cured adhesive (ASTM D4541, USA) and then pulling off the dolly by a steadily increasing force. The highest pulling force, representing the mechanical adhesion strength of ELP-Ni-P film, was recorded at the moment when ELP Ni-P film was detached from the Si wafer. The elemental analysis of Ni-P film before and after RTA was done by using the EDX, EMAX-ENERGY (Horiba) system model and the accelerating voltage was fixed at 15 kV. The operating time was fixed at 200 seconds for each examination.

### Availability of materials and data

The data and materials shown in this article are available and repeatable.

## Electronic supplementary material

Electronic Supplementary Information
