# Supplementary material for: Manipulating the adhesion of electroless nickel-phosphorus film on silicon wafers by silane compound modification and rapid thermal annealing
Source: Sci Rep. 2017 Aug 29;7:9656. doi: 10.1038/s41598-017-08639-x (PMC5574985; doi:10.1038/s41598-017-08639-x)
Supplement: Supplementary file 1 — Electronic Supplementary Information [file 41598_2017_8639_MOESM1_ESM.pdf]

Electronic Supplementary Information

Manipulating the adhesion of electroless nickel-phosphorus film on silicon wafers by silane compound modification and rapid thermal annealing

C. W. Hsu, W. Y. Wang, K. T. Wang, H. A. Chen and T. C. Wei

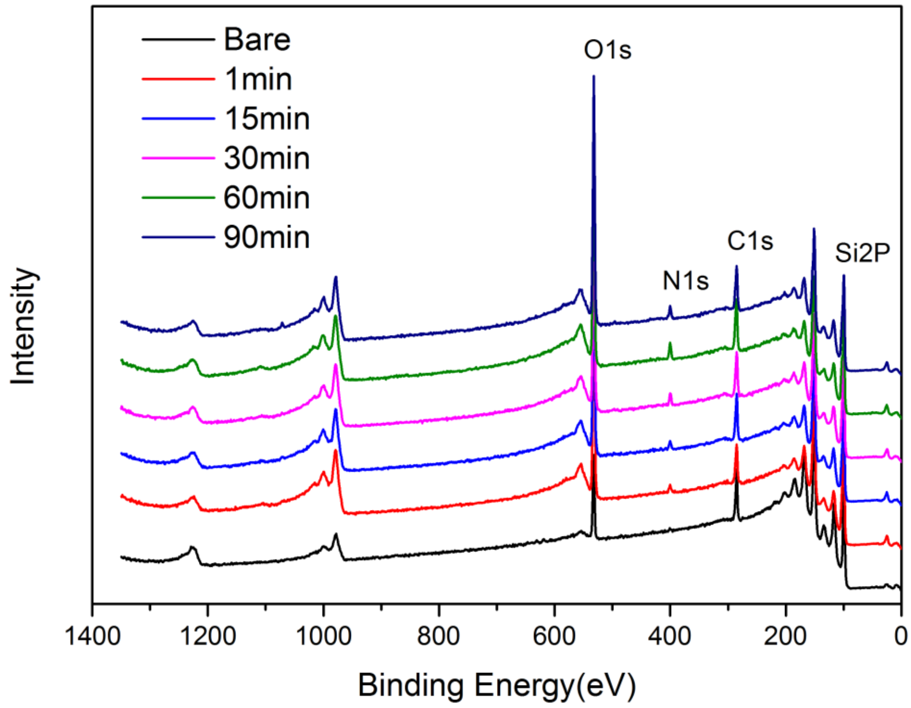

Figure S1. XPS wide spectrum of bare and various ETAS-modified Si wafers

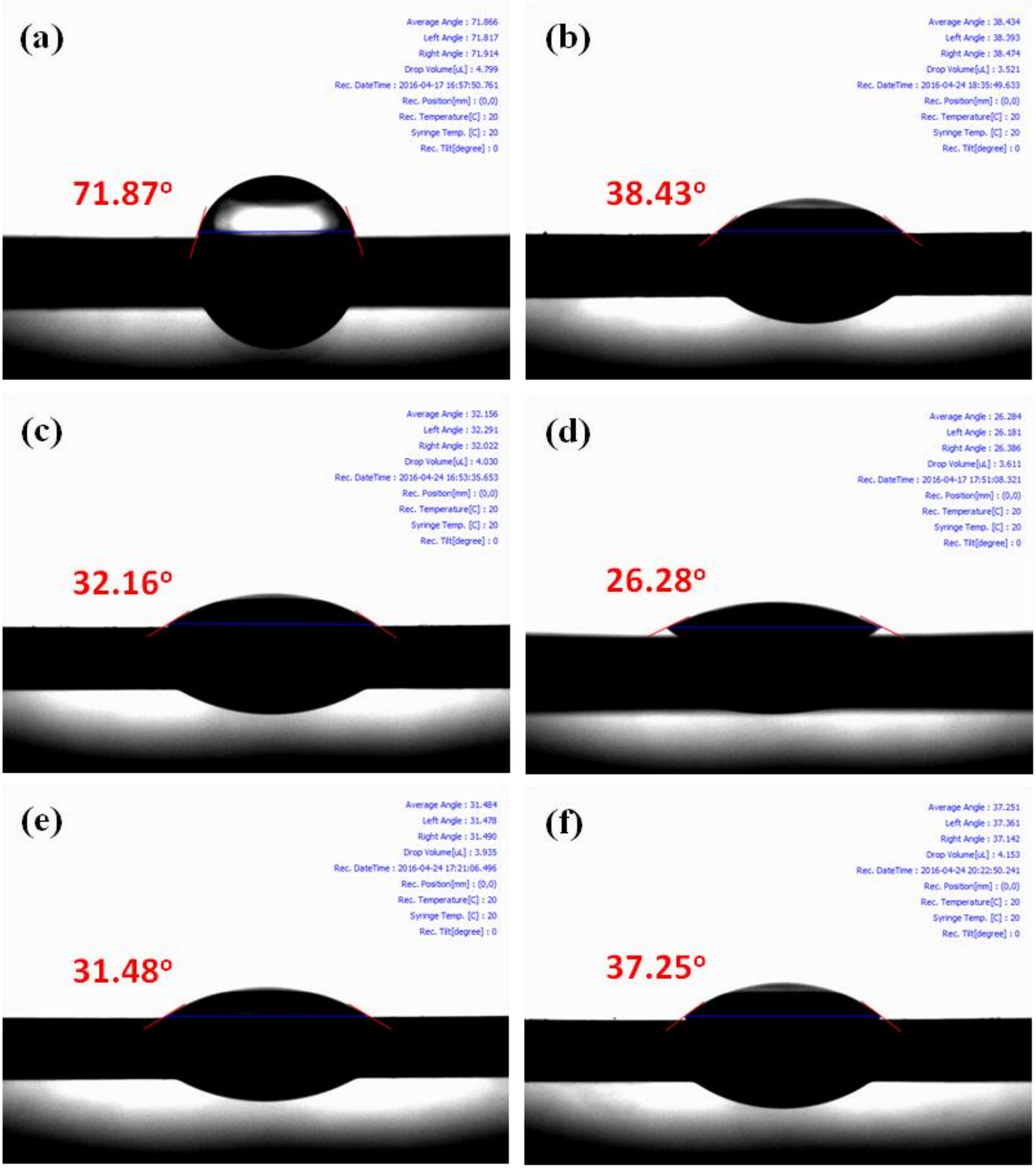

Figure S2. Representative WCA images of (a) bare wafer, (b) ETAS-1, (c) ETAS-15, (d) ETAS-30, (e) ETAS-60 and (f) ETAS-90.

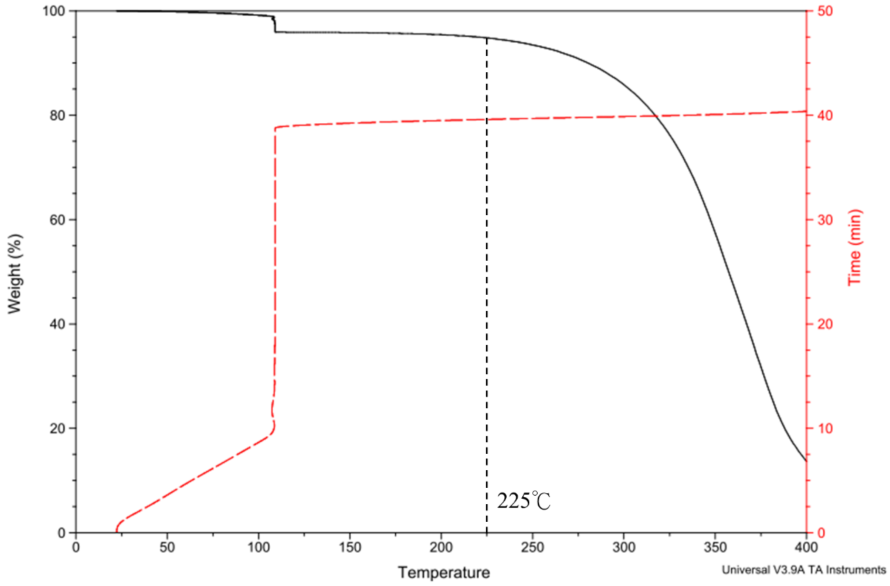

Figure S3. TGA curve of pure ETAS

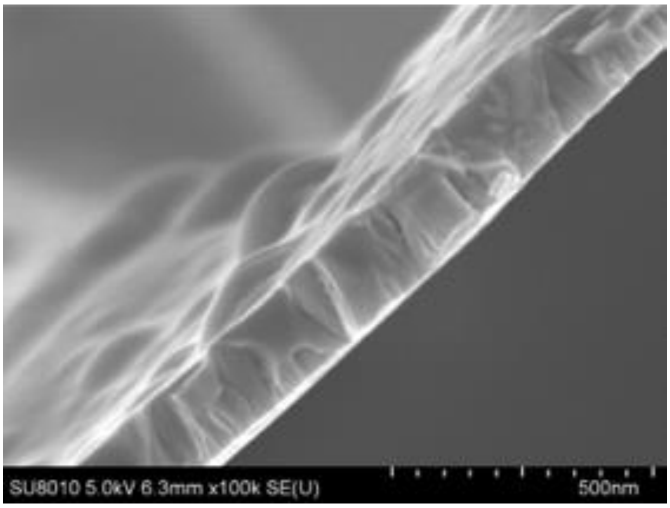

Figure S4. Typical ELP Ni-P film thickness (approximately 200nm) fabricated in this study.
